# Quality of Type II Diabetes Care in Primary Health Care Centers in Kuwait: Employment of a Diabetes Quality Indicator Set (DQIS)

**DOI:** 10.1371/journal.pone.0132883

**Published:** 2015-07-15

**Authors:** Dalia Badawi, Shadi Saleh, Nabil Natafgi, Yara Mourad, Kazem Behbehani

**Affiliations:** 1 Dasman Diabetes Institute, Kuwait City, Kuwait; 2 Department of Health Management and Policy, Faculty of Health Sciences, American University of Beirut, Beirut, Lebanon; 3 Department of Health Management and Policy, College of Public Health, University of Iowa, Iowa City, Iowa, United States of America; University of Glasgow, UNITED KINGDOM

## Abstract

Diabetes Mellitus is one of the major public health challenges, affecting more than 347 million adults worldwide. The impact of diabetes necessitates assessing the quality of care received by people with diabetes, especially in countries with a significant diabetes burden such as Kuwait. This paper aimed at piloting an approach for measuring Type II diabetes care performance through the use of a diabetes quality indicator set (DQIS) in primary health care. The DQIS for Kuwait was adapted from that developed by the National Diabetes Quality Improvement Alliance and the International Diabetes Federation. Five key care domains/measures were employed: (1) Blood glucose level measurement, (2) Cholesterol level measurement, (3) Blood pressure measurement, (4) Kidney function testing and (5) Smoking status check. The sample included the four major primary health care centers with the highest case load in Kuwait City, 4,241 patients in 2012 and 3,211 in 2010. Findings revealed the applicability and utility of employing performance indicators for diabetes care in Kuwait. Furthermore, findings revealed that many of the primary health care centers have achieved noteworthy improvement in diabetes care between 2010 and 2012, with the exception of smoking status check. The DQIS can help policymakers identify performance gaps and investigate key system roadblocks related to diabetes care in Kuwait.

## Introduction

**Fig 1:**
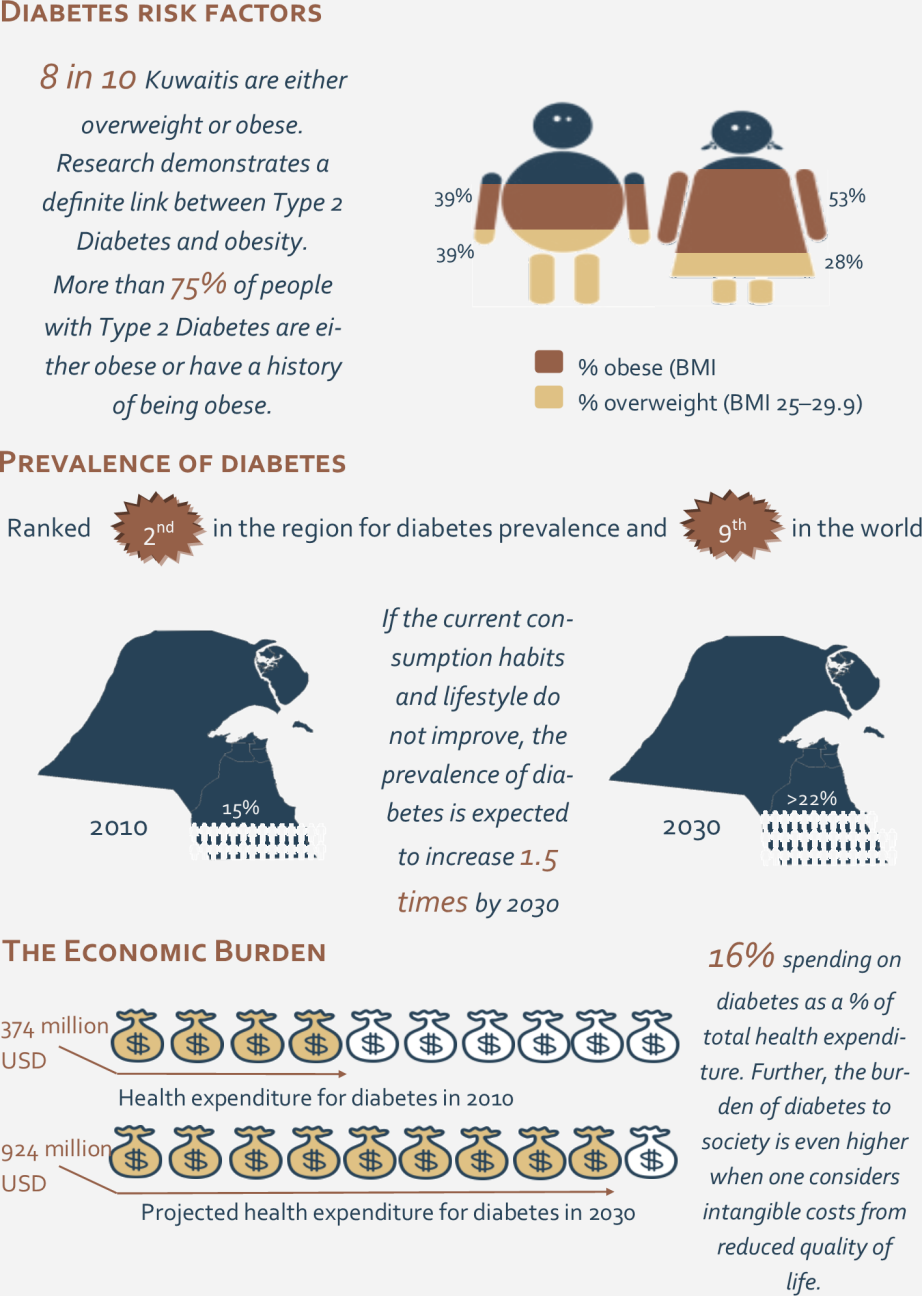
Key facts and figures on Diabetes in Kuwait. Data compiled from various sources: (i) The Impact of Main Chronic Conditions in Kuwait—Diabetes, Dasman Diabetes Institute; (ii) Musaiger, 2011, Overweight and Obesity in Eastern Mediterranean Region: Prevalence and Possible Causes, Journal of Obesity; (iii) International Diabetes Federation Diabetes Atlas 5th Edition.

Diabetes Mellitus is one of the major public health challenges affecting more than 347 million adults worldwide [1]. The disease is estimated to affect 439 million by the year 2030, representing a 69% increase in the number of affected adults in developing countries [2]. According to the International Diabetes Federation (IDF), the total number of people with diabetes in the Middle East and North Africa (MENA) region has reached 34.6 million (one in ten individuals has diabetes), and it is estimated to reach 67.9 million by the year 2035 [3]. Individuals diagnosed with diabetes are at an increased risk of developing several life-threatening health problems, including cardiovascular, kidney, nerve and eye disease, as well as pregnancy-related complications. Moreover, around 368,000 deaths reported in 2013 were associated with diabetes [3]. Kuwait ranks ninth in the world and second in the region in terms of diabetes prevalence. Estimates for 2013 reported 407,530 individuals between the age of 20 and 79 diagnosed with diabetes in Kuwait (Fig 1) [3].

In light of the impact of diabetes on communities, assessing the quality of care received by people with diabetes in any health care system is of paramount importance. In Kuwait, this is evolving as a priority for the Ministry of Health (MOH) that manages around 80% of healthcare services, while the remaining 20% are provided by governmental agencies and the private sector. Healthcare delivery lies within a three-tiered system: primary, secondary, and tertiary. There are six health regions; each equipped with a general hospital and several primary health care (PHC) centers (PHCC) [4]. A vigorous PHC infrastructure has been established in Kuwait with services offered at 92 primary health care centers [5]. Such services include family medicine, maternal and child care, diabetes care, dental care, and prevention and vaccination [4]. Diabetes care is provided in selected PHCCs through what is referred to as “diabetes mini-clinics”. Referral policies outline the second level of care provided at diabetes units in hospitals and, thereafter, to specialized centers such as the Dasman Diabetes Institute.

The emphasis on quality and patient safety assessment in health care organizations, which globally started with the publication of the Institute of Medicine report, ‘to err is human’, in 2000 [6], did not materialize until recently in most developing countries, including those in the MENA region. One approach that has been increasingly employed to assess quality is the use of quality or performance indicators. There is significant evidence on the effect of employment of such indicators when evaluating the care process and its impact on patient outcomes [7]. Policymakers need evidence to identify potential access or quality-of-care problems related to mostly secondary preventive health services, to plan specific interventions, and to evaluate how well these interventions achieve set goals of prevention [8]. In developing countries, however, the employment of such indicators is almost non-existent [9].

This paper presents the approach and findings from a demonstration project that was executed in Kuwait, aimed at selecting and employing process and outcome quality indicators to assess diabetes care at four major PHCCs (not diabetes care specialized centers) in Kuwait City. Efforts have been exerted over the past decade to strengthen the monitoring of the quality of diabetes care offered in Kuwait [10]. However, the additional contribution of this paper to earlier work is the assessment of outcome, not only process, measures of diabetes care. The overarching objective was to pilot this methodology/approach for expansion to all PHCC in Kuwait, with the ultimate goal of identifying and capitalizing on opportunities for improvement of the quality of diabetic care among PHCC in Kuwait.

## Materials and Methods

### Diabetes Quality Indicators Set

The Diabetes Quality Indicators Set (DQIS) was adapted from a performance measurement set for adult diabetes care developed by the National Diabetes Quality Improvement Alliance (NDQIA) and the Global Guidelines for Type II Diabetes of the International Diabetes Federation (IDF) [11,12]. Measures were agreed upon in consultation with different associations and agencies associated with diabetes and quality of care. The identification and selection of the specific set of quality indicators was done by a panel of experts from the Dasman Diabetes Institute, Kuwait Ministry of Health and PHC representatives, in addition to experts from the American University of Beirut. The selection was based on (i) evidence linking process measures to important clinical outcomes; (ii) feasibility of implementation; and (iii) applicability in the Kuwaiti health care setting.

Five key care domains/measures were identified to assess the quality of services and care that people with diabetes should have access to, and those are: blood glucose level measurement, cholesterol level measurement, blood pressure measurement, kidney function testing, and smoking status check. It is important to note that the data recorded on the electronic medical records (EMR) system on diabetes performance was not comprehensive. Hence, certain clinical performance indicators were not reported in the study.

Specifically, seven indicators were adopted to measure the PHC performance in relation to diabetes management, four of which are process indicators and three are outcome indicators:

*Process Indicators*

1. Glycosylated Hemoglobin (HbA1c) Management: Percentage of patients with one or more HbA1c tests annually
2. Cholesterol/Lipid Measurement: Percentage of patients with at least one low density lipoprotein (LDL) cholesterol test annually
3. Annual screening of nephropathy: Percentage of patients with at least one test for urinary micro-albumin during the measurement year; or who had evidence of medical attention for existing nephropathy. The urinary micro-albumin test is a urine test that measures the amount of albumin in the urine. If kidney damage has occurred, albumin will leak into the bloodstream and will be present in the urine.
4. Annual smoking status check: Percentage of patients whose smoking status was ascertained and documented annually

*Outcome Indicators*

1. HbA1c control: Percentage of patients with most recent HbA1c level >9.0% (poor control) [13]
2. LDL cholesterol control: Percentage of patients with most recent LDL <100 mg/dl
3. Blood pressure control: Percentage of patients with most recent blood pressure <140/90 mmHg

Estimation of indicators assumed that the denominator comprised clinically diagnosed people with diabetes (between the ages 18 and 75 years).

### Data sources and analytical approach

The sample included the four main PHCC in Kuwait City carrying the highest case load, three of which are governmental and one private. The total count of patients (between the ages of 18 and 75 years) diagnosed with type II diabetes at these centers (denominator) during the year 2012 was 4,241 patients, distributed as follows: Center 1 (1,049 patients), Center 2 (910 patients), Center 3 (1,008 patients), and Center 4 (1,274 patients). The total count of patients (between the ages of 18 and 75 years) diagnosed with diabetes at these centers during the year 2010 was 3,211 patients, distributed as follows: Center 1 (925 patients), Center 2 (558 patients), Center 3 (912 patients), and Center 4 (816 patients).

Data needed for assessment of indicators (numerators and denominators) was extracted from the integrated information system at these centers, a system that is linked to the EMR and is in the process of being rolled out to most PHCC in Kuwait. The computation of quality indicators entailed extraction of data from different administrative (patient count and enrollment) and/or clinical (procedures and test results) sources based on the type of measure. Measurement period derived from several sources including lab results (HbA1c, LDL, Microalbumin) and patient encounters/visits. Time frame of data presented in this report includes years 2010 and 2012, extending from January 1, 2010 to December 31, 2010 and January 1, 2012 to December 31, 2012, respectively. Missing data in the denominator (patients not recorded) were not available and hence not included in the analysis. As for instances where the numerator was not available, information on that case was not reported for the specific indicator.

Two international benchmarks were employed as a reference for the generated results: (1) the US-based National Committee for Quality Assurance (NCQA) Healthcare Effectiveness Data and Information Set (HEDIS) Effectiveness of Care Measures– 2010 National HMO Averages; and (2) the European United Kingdom (UK)-based Diabetes-UK State of the Nation 2012 England Report [13–14].

Ethical approval for the study was granted by the Institutional Review Board (IRB) at the American University of Beirut (AUB). All patient information was de-identified for the purpose of analysis.

## Results

### Glycosylated Hemoglobin (HbA1c) management and control

**Fig 2:**
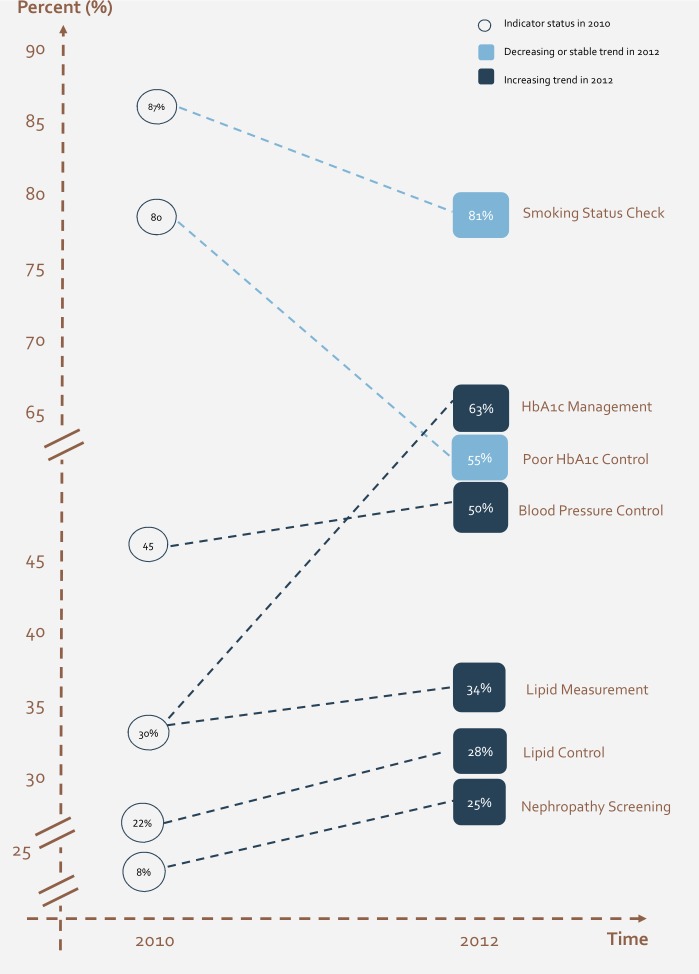
Trend of indicators fluctuation between 2010 and 2012.

Among the study sample of patients diagnosed with diabetes in the four centers, around 63% (2,679 patients) had received one or more HbA1c test during the measurement period in year 2012, as compared to only 30% of the sample of patients included in year 2010. Out of those who received at least one testing in 2012, 787 patients had the most recent HbA1c measure indicative of poor diabetes control (Table 1). The level of poor HbA1c control had decreased from that in year 2010 (80%) (Fig 2).

**Table 1:** Overall results for the Diabetes Quality Indicators Set (DQIS) for all PHC centers.

% 2012(2010)	HbA1c Management	Poor HbA1c Control*	Lipid Measurement	LDL Control	Nephropathy Screening	BP Control	Smoking Status Check
**Center 1**	74(30)	48(79)	54(38)	46(28)	33(11)	67(53)	95(93)
**Center 2**	47(25)	66(83)	13(26)	10(18)	18(7)	25(25)	57(71)
**Center 3**	67(32)	56(82)	30(26)	24(19)	26(6)	49(49)	86(87)
**Center 4**	63(32)	54(77)	36(27)	30(20)	25(8)	53(45)	81(91)
**Average**	63 (30)	55(80)	34 (30)	28 (22)	25 (8)	50 (45)	81(87)

*Poor HbA1c control measures indicate the percentage of patients with *poor glycemic control*, i.e. lower results reflects better control

**Fig 3:**
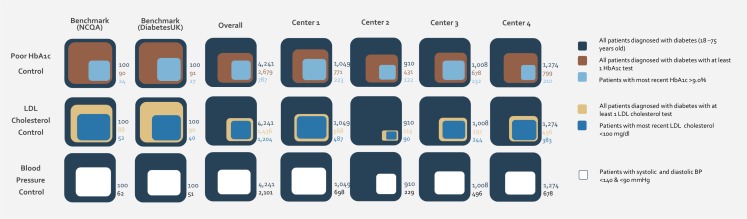
Control measures: Glycosylated Hemoglobin (HbA1c), Low-Density Lipoprotein (LDL) cholesterol, and blood pressure (2012)—comparison of PHC centers. The baseline of diabetic patients for benchmarks was set at 100 as a reference.

Comparing the performance of the four centers, one can observe that the highest level of HbA1c testing was recorded in Center 1 with 74% in 2012 (30% in 2010), as compared to the lowest level in Center 2 with 47% in 2012 (25% in 2010). The lowest degree of poor HbA1c control, in 2012, was observed in Center 1 (48%) and the highest in Center 2 (66%) (Fig 3).

### Cholesterol/Lipid level measurement and control

**Fig 4:**
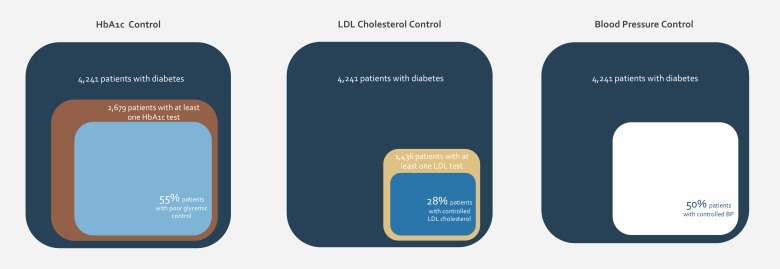
Control measures: Glycosylated Hemoglobin (HbA1c), Low-Density Lipoprotein (LDL) cholesterol, and blood pressure (2012)—comparison of overall measures.

Around 34% of the patients with diabetes in the study sample underwent an annual lipid measurement in 2012, as compared to 30% in 2010 (Table 1). The percentage of patients with good lipid control (most recent LDL cholesterol less than 100 mg/dl) in year 2012 was 28%, which represents a 27% improvement from year 2010 during which 22% of people with diabetes achieved good lipid control (Fig 4). The centers exhibited variation in performance with regards to annual lipid measurement, yet had similar results with regards to lipid control. The highest frequency of lipid measurement was recorded in Center 1 with 54% in 2012 (38% in 2010) and the lowest in Center 2 with 13% in 2012 (26% in 2010). Similarly, for good lipid control, the highest control rate, in year 2012, was recorded in Center 1 with 46% and the lowest in Center 2 with 10%.

### Annual screening of nephropathy

During the measurement period of 2012, only a quarter of the people with diabetes underwent a screening for nephropathy as part of their annual checks, as compared to 8% in year 2010 (Table 1). Some variation in the level of kidney function testing was observed across centers in 2012 with the highest rate being 33% in Center 1 and the lowest 18% in Center 2.

### Blood pressure control

With regards to blood pressure (BP), half of the people with diabetes included in the study demonstrated good control in 2012, as compared to a control rate of 45% in 2010 (Table 1). Comparison of PHCC reveals a variation in performance in regards to this indicator. The highest rate for good BP control was recorded in Center 1 at 67% and the lowest in Center 2 at 25%.

### Smoking status check

Overall, documentation of annual smoking status check for people with diabetes in 2012 was detected to be 81% (Table 1), with the highest rate of smoking status documentation being 95% in Center 1 and the lowest being 57% in Center 2. This indicator witnessed a 7.5% drop between 2010 and 2012.

## Discussion

The importance of assessing the quality of care received by people with diabetes in any health care system has been well-established [15–16]. More specifically, the employment of performance indicators, when evaluating the care process, has been associated with favorable impact on patient outcomes [7]. Such findings have driven the objectives of this study in assessing the applicability of indicators in the PHC setting of Kuwait through comparative analysis among major PHCC.

Findings of the study support the utilization of DQIS indicators in assessing the performance of the PHC sector in Kuwait City vis-à-vis diabetes care in comparison to international benchmarks of selected indicators. Data collected through the health information system that will soon be integrated in all PHCC across Kuwait can be employed to generate these indicators. The resulting findings from the DQIS can help define and assess the patterns and quality of diabetic care in PHCC in Kuwait. Moreover, the DQIS can aid policymakers in identifying performance gaps and further investigating key system roadblocks that are related to access and quality of diabetic care among PHCC in Kuwait. Experiences of other countries with the use of indicators for quality assessment of diabetes care have displayed similar favorable outcomes. For example, indicators adopted by the NDQIA have been utilized extensively in the U.S. to assess changes in outcomes of care over periods of time, and have proven effective in monitoring the improvement in the quality of diabetes care [17]. Similar efforts have been exerted by the Organization for Economic Cooperation and Development (OECD) towards using quality indicators adopted from that of the NDQIA in order to benchmark the performance of health care systems. Moreover, the Swedish National Diabetes Register (NDR) established in 1996 has been undergoing several updates and revisions in order to serve as a tool for quality control and benchmarking against nationally set targets [18].

Around 63% of patients with diabetes in this study sample had received at least one HbA1c test during the measurement period of 2012. Although there was a considerable improvement in the level of testing from year 2010 (more than doubling in value), compared to international benchmarks from the US (90%), UK (83%) and Italy (88%), Kuwait scored relatively lower with respect to annual HbA1c management [13–14]. The measurement of HbA1c is an essential indicator for optimal quality of diabetes care, as performance on this measure is reflective of how well steps of the care process are serving towards optimal glycemic control [3]. If incorporated within a comprehensive system of care in which patients receive all of the key care processes, regular HbA1c measurement may contribute to effective diabetes management. Indeed, the HbA1c check should be the most frequently carried out test for people with diabetes, conducted at around six month intervals for individuals with well-controlled diabetes [14].

Moreover, with regards to HbA1c control, 55.4% of people with diabetes displayed poor control. There had been a considerable enhancement in Hb1Ac control at the PHCC included in this study from year 2010 (around 80% poor control). The study findings also indicated an improvement in control of blood glucose among diabetics in Kuwait, as results obtained from an earlier study showed that around 66.7% of the studied population displayed poor control [19]. Moreover, the control observed from findings of this study placed Kuwait at a comparable status to other countries in the region, in terms of performance. Multiple studies that reviewed the quality of diabetes management in several GCC countries found that <50% of patients achieved target glycemic control [20–22]. Internationally, Kuwait compared favorably with countries such as Trinidad where 85% of people with diabetes displayed poor glycemic control [23]. However, in comparison to the US, where 28% of people with diabetes displayed poor glycemic control, Kuwait ranks low [13].

Data trending revealed the doubling of the HbA1c measurements (from 30% to 63%) between 2010 and 2012, and the decrease in the percentage of poorly controlled HbA1c from around 80% to 55%. This is indicative of enhanced compliance rates among patients and an improved systematic approach to monitor and control HbA1c rates. Although certain studies have noted a mild disassociation between adherence to measurement and quality outputs [24], it remains widely accepted that an association does exist between the two[25–27]. This is supported by the findings from this study where an increase in measurement rates was coupled with reduced poor glycemic control among diabetics.

Kuwait displayed poor performance in terms of cholesterol level measurement with only 34% of the people with diabetes tested for LDL cholesterol in 2012, and only a slight improvement in level of testing from year 2010. In comparison with other countries regionally and internationally, Kuwait scored the lowest. Indeed, annual lipid measurement was found to reach 50% in Spain and Australia, 58% in Abu Dhabi, 64% in New-Zealand, 67% in Thailand, and 70% in Italy [28]. Since lipid measurement is usually coupled to HbA1c measurement for people with diabetes, international benchmarks from USA and UK also indicate that on average around 90% of the people with diabetes have their cholesterol checked (similar to HbA1c benchmarks), which further shows that Kuwait lags behind [13–14].

Moreover, only 28% of the total diabetic population in the study achieved good lipid control. Such results are comparable to those of previous studies on the GCC countries that reported rates of good lipid control among approximately 30–50% of patients [20]. A study in Dubai showed that the proportion of people with diabetes with desirable LDL-cholesterol level ranged from 20.8% to 33.6% between 2003 and 2005. By comparison, 23% of patients in the USA [29] and 52.8% [30] in Australia achieved ADA targets in 2004 [29]. In the UK, as well, the percentage of people achieving their target cholesterol is only around 40% [14]. Suboptimal control of cholesterol may be attributed to the low rate of annual cholesterol measurement among people with diabetes. Enhanced screening/measurement may serve as the gateway to highlight the need to monitor individuals and consequently improving control rates. This is mirrored by the fact that despite the slight increase in the annual lipid measurement (from 30% in 2010 to 34% in 2012), the lipid control level among those who had their annual cholesterol being checked witnessed an increase of 27% (from 22% in 2010 to 28% in 2012). On another note, the enhanced lipid control level reflects the commitment of diabetic individuals in regulating their blood cholesterol level. Poor cholesterol control among diabetics may result in an increased risk for cardiovascular diseases, including heart attacks and strokes [31]. Moreover, the World Heart Federation ensures that by controlling the level of blood lipids, cardiovascular disease complications can be reduced by 20% to 50% [32].

The rate of BP measurement among people with diabetes in the centers was not calculated for 2012; the rate at baseline year measurement did not exceed 50%. International benchmarks indicate that this number compares favorably to the UK (51%) and USA (61%) standards [13–14]. BP control showed a slight improvement of 11% increase between 2010 and 2012. Several factors can explain such less-than-optimal level of BP control, such as inadequate treatment regimens, inability to detect high BP in the clinic setting, counter-indication of antihypertensive medications, patients’ in adherence to prescribed medication regimens, as well as lifestyle choices [33]. Similar to high blood cholesterol levels, elevated BP subjects individuals and more so those diagnosed with diabetes, to an increased risk of complications such as cardiovascular diseases and strokes [14].

In 2012, only 25% of the people with diabetes underwent kidney function testing as part of their annual checks. While more patients have received testing of their kidney function in 2012 than in 2010 (8%), the rate is still considered very low in comparison to international benchmarks of 70% in UK and 89% in USA [13–14]. It is important to note that the rate detected for urinary microalbumin is similar to that of earlier studies conducted in Kuwait [10]. Kidney failure is one of the most severe and life-threatening complications of diabetes. Hence, it is alarming that such a small percentage of patients are receiving kidney function testing. Such findings in Kuwait may be indicative of insufficient referral by providers for kidney function testing. Thus, this highlights the dire need for regular kidney function testing (at least once a year) to be reinforced as part of the key care processes and patients’ annual review check, as per recognized standards of care [14].

As reflected earlier in the paper, being diagnosed with diabetes already exposes individuals to an increased risk of heart disease and stroke, in addition to a number of other complications, and smoking further amplifies this risk [31]. Patients with diabetes who are smokers must therefore receive advice and support on how to quit smoking [14]. Moreover, monitoring of smoking status should be carried out and recorded at least once a year [14]. In Kuwait, the average rate of annual smoking status check was at 81%. Compared to England, where the annual level of recording smoking status reaches on average around 85% [14], the current performance in PHCC of Kuwait ranked comparable. However, performance had dropped from year 2010, and thus there remains a need for interventions to maintin a higher level of annual smoking status checks amongst people with diabetes.

The study has few limitations that are worth mentioning. First, although the four main PHCC in Kuwait city were included in the study, one cannot ensure that results in other centers would have been similar. However, a certain degree of generalizability can be assumed given the volume of patients channeled through these centers. Second, the data recorded on the EMR system on diabetes performance was not comprehensive. Certain clinical performance indicators were not recorded in the EMR and consequently were not reported for all people with diabetes in these centers. There is no reason to believe that there is are systematic factors affecting the rate of missing clinical data among centers, or in comparison with national estimates. However, this remains a limitation in the absence of thorough data and analysis of missing information.

In conclusion, DQIS can serve as an evidence-based tool for health care professionals, health care organizations and policy makers in enhancing diabetes care. This is particularly relevant in Kuwait given the burden of diabetes and the need to employ approaches to assess and enhance diabetes care. However, the expansion of DQIS, or a more comprehensive version of it, to all primary health care centers is key to witnessing system level improvements.
